# High-Throughput Drug Screening in Chondrosarcoma Cells Identifies Effective Antineoplastic Agents Independent of IDH Mutation

**DOI:** 10.3390/ijms252313003

**Published:** 2024-12-03

**Authors:** Luyuan Li, Lily Hashemi, Josiane Eid, Wensi Tao, Leticia Campoverde, Amy Yu, Ammad Ahmad Farooqi, Hassan Al-Ali, Gina D’Amato, Francis Hornicek, Zhenfeng Duan, Ines Lohse, Jonathan Trent

**Affiliations:** 1 Department of Medicine, Division of Medical Oncology, University of Miami Miller School of Medicine, Miami, FL 33136, USA; 2 Sylvester Comprehensive Cancer Center, University of Miami Health System, Miami, FL 33136, USA; 3 College of Science, Northeastern University, Boston, MA 02115, USA; 4 Department of Radiation Oncology, University of Miami Miller School of Medicine, Miami, FL 33136, USA; 5 The University of Texas MD Anderson Cancer Center, Houston, TX 77030, USA; 6 Department of Medicine, Johns Hopkins School of Medicine, Baltimore, MD 21205, USA; 7 Institute of Biomedical and Genetic Engineering, Islamabad 44000, Pakistan; 8 Department of Neurosurgery, University of Miami Miller School of Medicine, Miami, FL 33136, USA; 9 Miami Project to Cure Paralysis, University of Miami Miller School of Medicine, Miami, FL 33136, USA; 10 Katz Family Division of Nephrology and Hypertension, Department of Medicine, University of Miami Miller School of Medicine, Miami, FL 33136, USA; 11 Peggy and Harold Katz Family Drug Discovery Center, University of Miami Miller School of Medicine, Miami, FL 33136, USA; 12 Frost Institute for Data Science and Computing, University of Miami Miller School of Medicine, Miami, FL 33136, USA; 13 Department of Orthopedics, University of Miami Miller School of Medicine, Miami, FL 33136, USA; 14 Center for Therapeutic Innovation, University of Miami Miller School of Medicine, Miami, FL 33136, USA; 15 Department of Orthopedic Surgery, University of Pittsburgh, Pittsburgh, PA 15213, USA

**Keywords:** chondrosarcoma, high-throughput screening, anticancer compounds, IDH mutation, synergy

## Abstract

The term chondrosarcoma refers to a rare and heterogeneous group of malignant cartilaginous tumors that are typically resistant to chemotherapy and radiotherapy. Metastatic chondrosarcoma has a poor prognosis, and effective systemic therapies are lacking. Isocitrate dehydrogenase (IDH) mutations represent a potential therapeutic target, but IDH inhibitors alone have shown limited clinical efficacy to date. Although the role of conventional chemotherapy is still subject to debate, some evidence suggests it may provide therapeutic benefits in advanced cases. In this study, we aimed to identify effective compounds for combination therapy in chondrosarcoma. Using high-throughput screening, we evaluated a panel of anticancer agents in IDH1-mutant chondrosarcoma cell lines and their mutant IDH1 knockout derivatives. The top 20 most potent compounds were identified across all cell lines, irrespective of IDH mutation status. Representative drugs selected for further investigation included docetaxel, methotrexate, panobinostat, idarubicin, camptothecin, and pevonedistat. These drugs inhibited colony formation, induced apoptosis and cell cycle arrest, and exhibited synergistic antitumor activity in two-drug combinations. In conclusion, we identified several highly effective agents with potent anti-tumor activity in chondrosarcoma cells, independent of IDH mutation status. These agents represent promising candidates for chondrosarcoma therapy and warrant further preclinical investigation and potential inclusion in clinical trials.

## 1. Introduction

The term chondrosarcoma refers to a rare and heterogeneous group of malignant bone tumors arising from cartilage-producing cells. The estimated annual incidence of chondrosarcoma in the United States is about 1 in 200,000 [1,2]. It is the second most common type of bone sarcoma, following osteosarcoma, and represents more than 20% of primary bone malignancies [1,3]. Chondrosarcoma most commonly affects adults, though it can occur at any age, with the proximal femur and pelvic region being the most frequent sites of origin [1,3]. Conventional primary chondrosarcoma is the most prevalent subtype, accounting for approximately 85% of cases, while rarer variants include secondary chondrosarcoma (which arises from benign precursors), as well as dedifferentiated, periosteal, mesenchymal, and clear cell chondrosarcoma [1,3].

Chondrosarcoma is known for its poor response to traditional chemotherapy and radiotherapy, possibly because of the low mitotic activity of the tumor cells and restricted drug penetration due to the abundant extracellular matrix and poor vascularity [1,3]. However, the role of chemotherapy in the treatment of chondrosarcoma remains controversial. While clinical data on the efficacy of chemotherapy are scarce, some studies suggest it offers limited, yet non-negligible benefit, particularly in advanced cases. The greatest efficacy has been observed in mesenchymal and dedifferentiated subtypes [4,5]. Currently, surgical resection is the primary treatment for localized lesions, while radiotherapy is typically reserved for treating inoperable micro-lesions or for palliative management of residual local disease [3,6]. The prognosis of patients with chondrosarcoma is generally poor, largely due to local invasion and distant metastasis, most commonly of the lungs and occasionally of the liver, kidney, and brain [7,8]. The 5-year survival rate for chondrosarcoma patients varies by tumor subtype; for those with dedifferentiated chondrosarcoma, the survival rate is typically less than 25%, due to early and widespread metastasis [9,10,11]. Given the limited treatment options and poor prognosis, there is an urgent need to develop novel, effective therapeutic strategies to improve patient outcomes.

Gene mutations are commonly observed in cancers, and in some cases, they serve as the primary drivers of tumorigenesis. Multiple mutations, both germline and somatic, have been identified in chondrosarcoma tumors. The most common genetic alterations in chondrosarcoma are mutations in isocitrate dehydrogenase (IDH) 1 and 2. IDH1 and IDH2 mutations occur in approximately 50–75% of chondrosarcoma cases, with the prevalence varying by tumor subtype [12,13]. These mutations play an important role in the diagnosis of dedifferentiated chondrosarcoma and help differentiate chondrosarcoma from chondroblastic osteosarcoma [14]. IDH1 and IDH2 are enzymes involved in the tricarboxylic acid (TCA) cycle, where normal IDH proteins convert isocitrate to α-ketoglutarate (α-KG). In contrast, the mutant IDH proteins convert α-KG to the oncometabolite D-2-hydroxyglutarate (D-2HG) [15,16]. The role of IDH mutations in chondrosarcoma remains incompletely understood. While IDH mutations are thought to play a key role in the initiation of cartilage tumor development, their involvement in later stages of tumor progression is still unclear. In our previous study, we used the CRISPR/Cas9 system to knock out mutant IDH1 in two chondrosarcoma cell lines. This genetic modification led to a significant reduction in D-2HG production and tumorigenicity in the mutant IDH1 knockout (KO) cells [17,18]. Moreover, the loss of mutant IDH1 resulted in decreased chondrosarcoma tumor formation and D-2HG production in a xenograft model [17]. These findings suggest that IDH mutations could serve as a potential therapeutic target for chondrosarcoma. However, early clinical trial results focusing on IDH inhibitors alone have shown modest efficacy in treating chondrosarcoma, suggesting that combination therapy may be a more effective approach for future treatment strategies [19,20].

In this study, we aimed to identify potential therapeutic agents for chondrosarcoma by screening a library of anticancer compounds, with a particular focus on those that could serve as candidates for future combination therapies. We further assessed the effects of selected representative agents on apoptosis and cell cycle progression. Additionally, we evaluated the synergistic effects of two-drug combinations from the selected drugs in chondrosarcoma cells. Finally, we investigated whether depletion of mutant IDH could enhance the sensitivity of chondrosarcoma cells to any of the screened compounds.

## 2. Results

### 2.1. Drug Sensitivity Testing in Chondrosarcoma Cells

**Figure 1:**
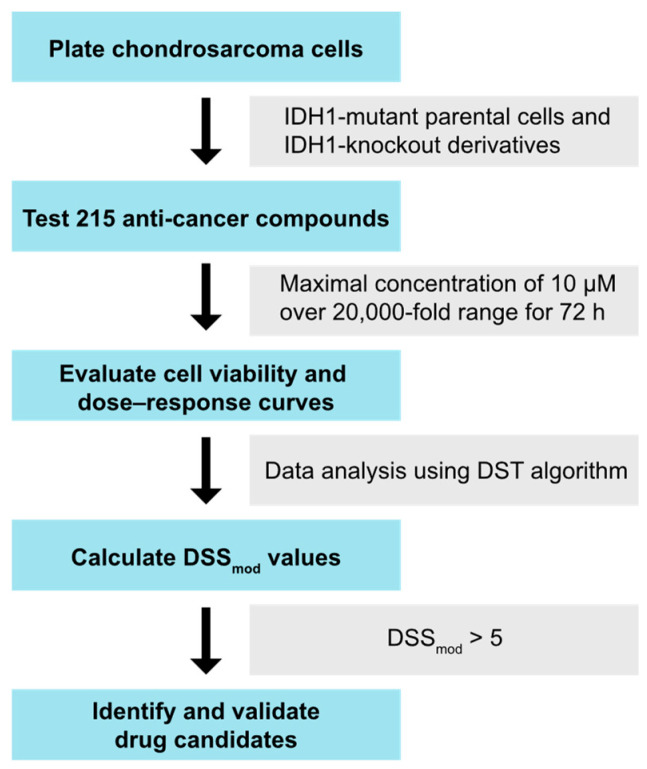
Drug sensitivity testing workflow. A total of 215 anticancer drugs were tested in chondrosarcoma cell lines JJ012 and HT1080, as well as their respective mutant IDH1 KO derivatives, J14 and H2. Each drug was dissolved in 100% DMSO, starting at a maximum concentration of 10 μM and then diluted across a 20,000-fold concentration range. A total of 1000 cells were seeded per well in 384-well microtiter plates. After 72 h of treatment, cell viability was assessed, and dose-response curves were generated. The DST algorithm was used to calculate the DSS_mod_ value for each drug. Drugs with a DSS_mod_ value greater than 5 were considered to show significant cancer cell-killing activity.

**Figure 2:**
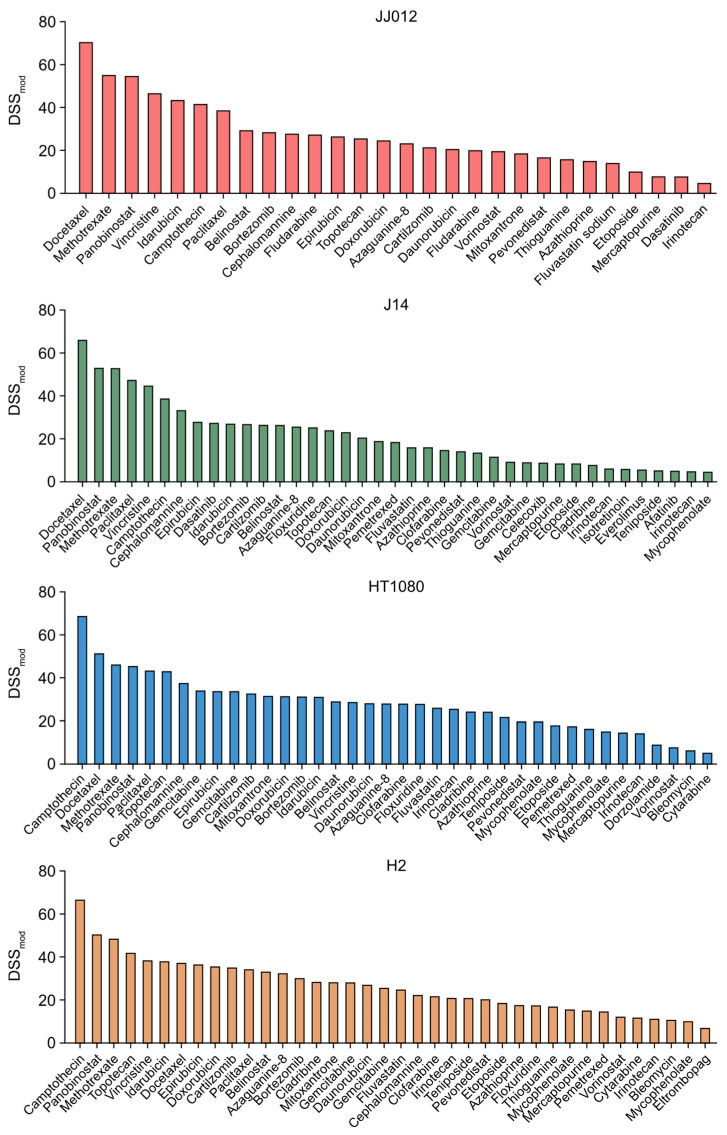
DSS_mod_ profiling in chondrosarcoma cell lines. The DSS_mod_ profile for each cell line shows compounds with a DSS_mod_ value greater than 5, indicating significant cancer cell-killing activity.

As outlined in the diagram in Figure 1, drug sensitivity testing (DST) was performed on chondrosarcoma cell lines JJ012 and HT1080, along with their respective mutant IDH1 knockout derivatives, J14 and H2, using an established compound library [21]. As shown in Table 1, the library includes 215 anticancer drugs commonly used in modern cancer therapy, whose mechanisms of action span a wide range of cancer-related pathways and molecular targets. The DSS_mod_ profile for each cell line shows compounds that displayed significant cancer cell-killing activity above the threshold (Figure 2). In JJ012 cells, 28 drugs were identified, with docetaxel (DSS_mod_: 70.9), methotrexate (DSS_mod_: 55.6), and panobinostat (DSS_mod_: 55.0) ranking as the top three compounds. In J14 cells, 39 drugs were identified, with docetaxel (DSS_mod_: 66.5), panobinostat (DSS_mod_: 53.5), and methotrexate (DSS_mod_: 53.4) being the top three hits. In HT1080 cells, 38 drugs were identified, with camptothecin (DSS_mod_: 69.2), docetaxel (DSS_mod_: 51.8), and methotrexate (DSS_mod_: 46.6) leading the list. Similarly, in H2 cells, 38 drugs were identified, with camptothecin (DSS_mod_: 67.1), panobinostat (DSS_mod_: 50.9), and methotrexate (DSS_mod_: 48.9) as the top three compounds.

The compound library includes 215 anti-cancer drugs commonly used in modern cancer therapy, classified according to their mechanisms of action where available. Notably, some compounds appear in multiple variations during screening, serving as internal controls, but are not listed as individual compounds in the table.

Significant similarities in drug sensitivity profiles were observed across all four cell lines, irrespective of IDH mutation status. Based on these results, we focused on the 20 compounds that demonstrated high cancer cell-killing activity in all four cell lines, as indicated by their DSS_mod_ values, though the magnitude of response varied slightly between cell lines. These compounds were classified according to their known mechanisms of action, including antimitotics, antimetabolites, histone deacetylase (HDAC) inhibitors, antitumor antibiotics/anthracyclines, topoisomerase 1/2 inhibitors, and a NEDD8-activating enzyme (NAE) inhibitor. The most potent compounds from each class—docetaxel, methotrexate, panobinostat, idarubicin, camptothecin, and pevonedistat—were selected for further investigation. Detailed DSS_mod_ values for the 20 compounds in each cell line are provided in Table 2, while the half-maximal effective concentrations (EC_50_) for the six representative drugs are shown in Table 3.

### 2.2. Validation of Drug Sensitivity Testing Results

**Figure 3:**
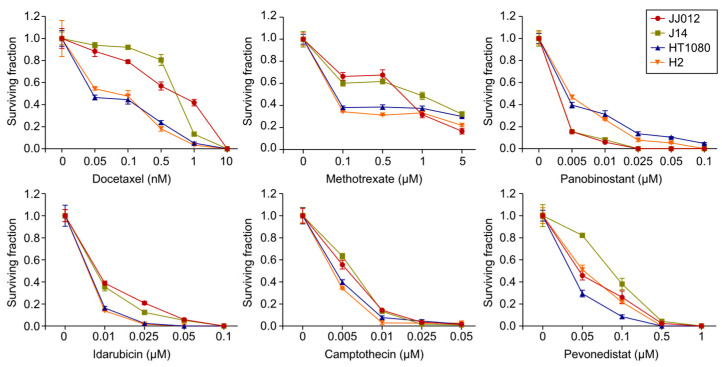
Validation of drug sensitivity testing results. Docetaxel (2 h), methotrexate (72 h), panobinostat (72 h), idarubicin (2 h), camptothecin (24 h), and pevonedistat (24 h) were evaluated via clonogenic assays to determine their effects on cell survival in chondrosarcoma cells.

To validate the results of the drug sensitivity testing, we evaluated the growth-inhibitory effects of the six selected drug candidates—docetaxel, methotrexate, panobinostat, idarubicin, camptothecin, and pevonedistat—on cell survival in the four chondrosarcoma cell lines using clonogenic assays. Drug concentrations and treatment durations were determined based on data from ATP-based cell viability assays conducted during the drug screening, as well as relevant literature on similar treatments in other cell types. Specifically, we focused on concentration ranges around the EC_50_ values obtained from the screening assays to ensure that meaningful differences could be detected between experimental and control groups. When treated with a range of doses (0.05 nM–5µM) at specific time points, the six drugs effectively inhibited colony formation in all four cell lines compared to the DMSO-treated vehicle controls (Figure 3). Notably, treatment with 10 nM docetaxel (2 h), 0.1 µM panobinostat (72 h), 0.1 µM idarubicin (2 h), 0.05 µM camptothecin (24 h), and 1 µM pevonedistat (24 h) resulted in complete inhibition of colony formation in all four cell lines. In contrast, methotrexate exhibited a different pattern: treatment with 5 µM (72 h) inhibited colony formation by 65–85% across the four cell lines, with the inhibitory effect plateauing between 0.1 and 0.5 µM in JJ012 and J14 cells, and between 0.1 and 1 µM in HT1080 and H2 cells.

### 2.3. The Selected Drugs Induced Apoptosis in Chondrosarcoma Cells

To assess the cytotoxic effects of the six selected drug candidates, we evaluated their ability to induce apoptosis in JJ012 and HT1080 cells using the concentrations and time points established in the clonogenic assays. Apoptosis was assessed via flow cytometry with Annexin V and propidium iodide (PI) staining. Annexin V detects both early and late stages of apoptosis, while PI staining identifies late apoptotic or necrotic cells with compromised membrane integrity. This method allowed us to differentiate between three distinct cell states:

1. Living cells (Annexin V and PI-negative).
2. Early apoptotic cells (Annexin V-positive and PI-negative).
3. Late apoptotic cells (Annexin V- and PI-positive).

**Figure 4:**
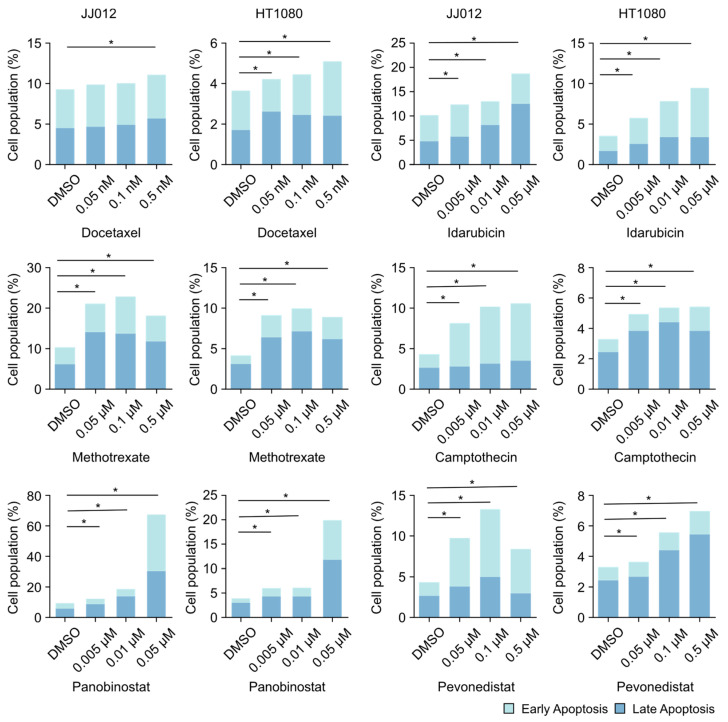
Docetaxel (2 h), idarubicin (2 h), methotrexate (72 h), camptothecin (24 h), panobinostat (72 h), and pevonedistat (24 h) induced apoptosis in chondrosarcoma cells. Total (early and late stages) apoptosis was statistically analyzed (* *p* < 0.05).

Flow cytometric analysis revealed that all six drugs induced significant apoptosis in both cell lines (*p* < 0.05, Figure 4). Among these, the most pronounced effect was observed for panobinostat (0.05 µM, 72 h), which induced 68% apoptosis in JJ012 cells and 20% in HT1080 cells, with notable increases in both early and late apoptotic populations compared to DMSO controls. In contrast, treatment with docetaxel (0.5 nM, 2 h) induced only a modest increase in apoptosis, with less than a 2% increase in total apoptosis in both JJ012 and HT1080 cells compared to controls. These results suggest that all six drugs, particularly panobinostat, are capable of inducing apoptosis in chondrosarcoma cells, underscoring their potential as therapeutic agents for this malignancy.

### 2.4. The Selected Drugs Induced Cell Cycle Arrest in Chondrosarcoma Cells

**Figure 5:**
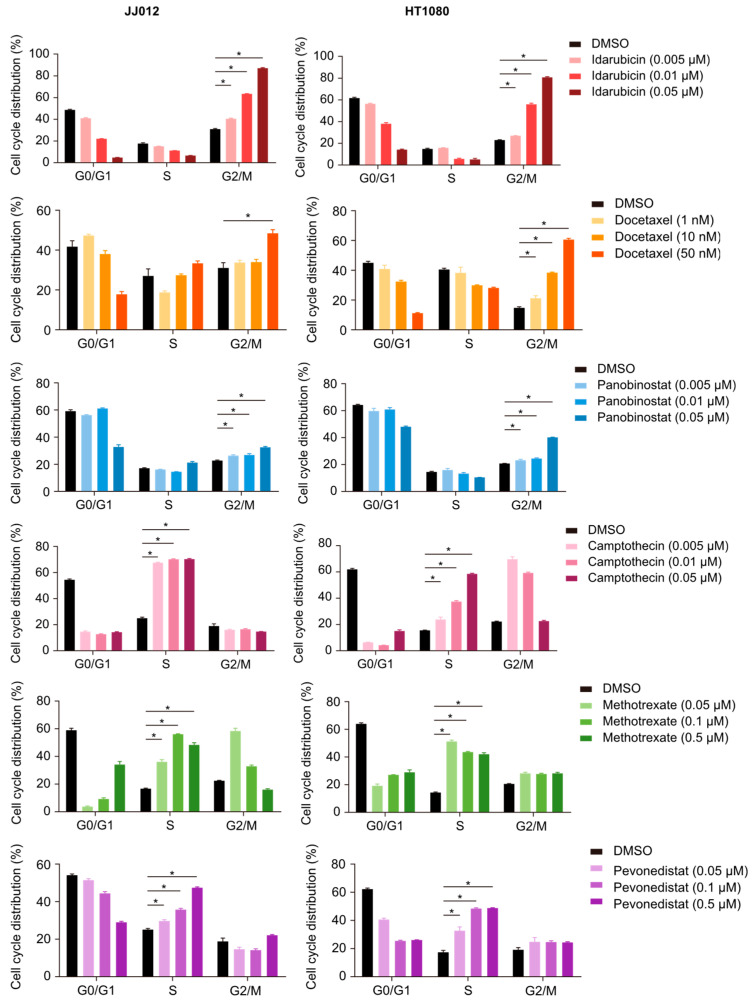
Docetaxel (2 h), idarubicin (2 h), methotrexate (72 h), camptothecin (24 h), panobinostat (72 h), and pevonedistat (24 h) induced cell cycle arrest in chondrosarcoma cells. * *p* < 0.05.

In addition to apoptosis analysis, we evaluated the effects of the six selected drug candidates on cell cycle progression by flow cytometry using PI staining. The results, which are shown in Figure 5, demonstrate significant alterations in the cell cycle profiles of both JJ012 and HT1080 cells following treatment with each drug, compared to DMSO-treated controls (*p* < 0.05). Notably, idarubicin, docetaxel, and panobinostat primarily induced cell cycle arrest at the G2/M phase in both cell lines. Among these, idarubicin (0.05 µM, 2 h) induced the most pronounced G2/M phase arrest, with 87% of JJ012 cells and 81% of HT1080 cells accumulating in this phase. Docetaxel (50 nM, 2 h) also resulted in G2/M phase arrest in 49% of JJ012 cells and 61% of HT1080 cells. Similarly, panobinostat (0.05 µM, 72 h) caused G2/M phase arrest in 32% of JJ012 cells and 40% of HT1080 cells. In contrast, camptothecin, methotrexate, and pevonedistat primarily induced cell cycle arrest in the S phase. Among these, camptothecin (0.05 µM, 24 h) induced the most significant S phase arrest, with 70% of JJ012 cells and 59% of HT1080 cells accumulating in this phase. Methotrexate (72 h) also resulted in S phase arrest in 56% of JJ012 cells (0.1 µM) and 51% of HT1080 cells (0.05 µM). Similarly, pevonedistat (0.5 µM, 24 h) caused S phase arrest in 47% of JJ012 cells and 49% of HT1080 cells. Taken together, these results demonstrate that the six drugs not only induce apoptosis but also significantly affect cell cycle progression, causing either G2/M or S phase arrest. These findings further highlight the potential of these drugs as promising therapeutic agents for chondrosarcoma.

### 2.5. The Selected Drugs Exerted Synergistic Antitumor Effects in Chondrosarcoma Cells

**Figure 6:**
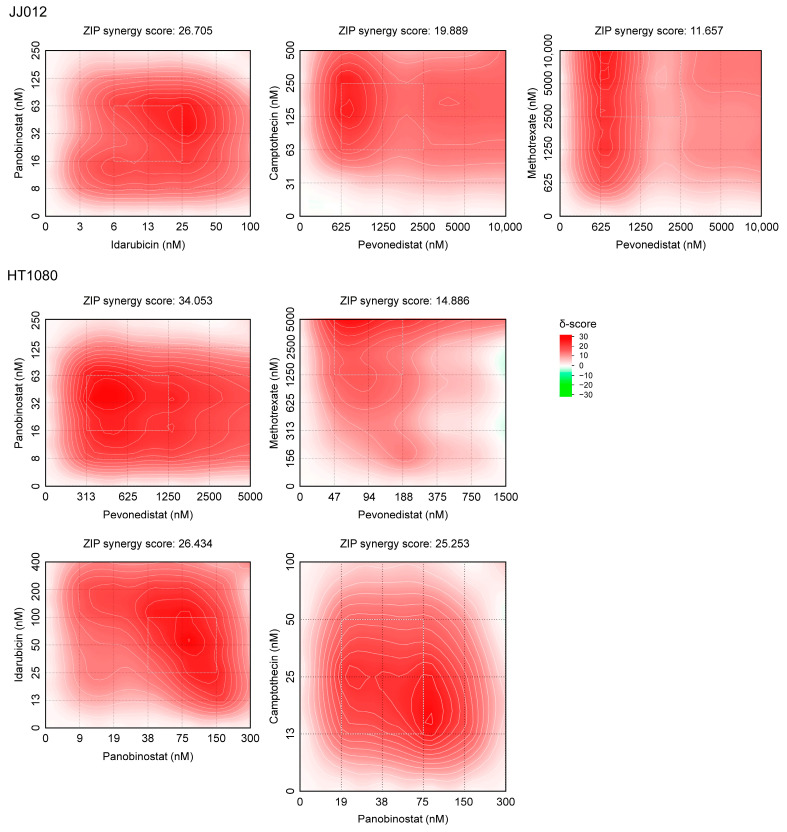
Two-dimensional synergy map showing the synergistic effects of two-drug combinations in chondrosarcoma cells (ZIP score > 10). Rectangles highlight the most synergistic areas.

Clinical practice has shown that combination chemotherapy often leads to higher overall response rates and complete remission compared to sequential single-agent treatments. In this study, we assessed the synergistic effects of two-drug combinations composed of the six selected drugs using the SynergyFinder 3.0 platform. In JJ012 cells, two-dimensional synergy maps revealed significant synergy between panobinostat and idarubicin, pevonedistat and camptothecin, and pevonedistat and methotrexate, with ZIP scores of 26.705, 19.889, and 11.657, respectively (Figure 6). The most synergistic regions in the synergy maps were 16–63 nM of panobinostat combined with 6–25 nM of idarubicin, 625–2500 nM of pevonedistat combined with 63–250 nM of camptothecin, and 625–2500 nM of pevonedistat combined with 2500–10,000 nM of methotrexate.

In HT1080 cells, synergistic effects were also observed for combinations of panobinostat and pevonedistat, pevonedistat and methotrexate, panobinostat and idarubicin, and panobinostat and camptothecin, with ZIP scores of 34.053, 14.886, 26.434, and 25.253, respectively (Figure 6). The most synergistic areas in the synergy map were 16–63 nM of panobinostat combined with 313–1250 nM of pevonedistat, 47–188 nM of pevonedistat combined with 1250–5000 nM of methotrexate, 38–150 nM of panobinostat combined with 25–100 nM of idarubicin, and 19–75 nM of panobinostat combined with 13–50 nM of camptothecin. Notably, the combinations of panobinostat and idarubicin, as well as pevonedistat and methotrexate, exhibited synergistic effects in both cell lines, although the latter combination required higher concentrations to achieve effective synergy. Moreover, panobinostat and pevonedistat were the drugs that most frequently exhibited synergistic interactions with other drugs, highlighting their strong potential for use in combination-based therapeutic regimens for chondrosarcoma.

### 2.6. Drug Sensitivity Analysis in Mutant IDH Knockout Cells Compared to Parental Cells

**Figure 7:**
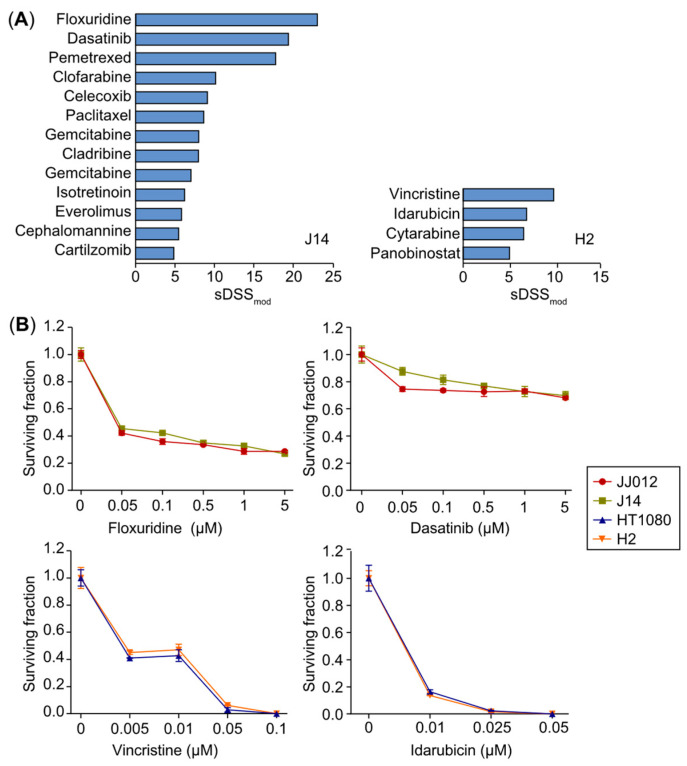
Drug sensitivity analysis in mutant IDH knockout cells compared to parental cells. (**A**) The sDSS_mod_ profiling for J14 and H2 cells shows drugs with a score greater than +5, indicating favorable effects on mutant IDH1 KO cells. (**B**) Validation of the top two hit drugs from J14 and H2 profiling using clonogenic assays. Top: The effects of floxuridine (48 h) and dasatinib (24 h) on cell survival were evaluated in J14 and JJ012 cells. Bottom: The effects of vincristine (2 h) and idarubicin (2 h) on cell survival were evaluated in H2 and HT1080 cells.

IDH mutations represent an attractive therapeutic target in chondrosarcoma. However, early clinical data have shown that IDH inhibitors alone have limited effect, suggesting the need for alternative combination strategies. To explore whether depletion of mutant IDH sensitizes chondrosarcoma cells to compounds from our drug screening library, we performed a drug sensitivity analysis in mutant IDH1 KO clones (J14 and H2), comparing their responses with those of the parental cells. The sDSS_mod_ profile for each cell line was generated, displaying all drugs with a score greater than +5 (indicating favorable effects on KO cells). In J14 cells, 13 drug candidates were identified, with floxuridine (sDSS_mod_: 23.2) and dasatinib (sDSS_mod_: 19.6) as the top two hits (Figure 7A). In H2 cells, four candidates were identified, with vincristine (sDSS_mod_: 9.8) and idarubicin (sDSS_mod_: 6.9) as the top two hits (Figure 7A).

To validate these results, we assessed the effects of floxuridine (48 h) and dasatinib (24 h) on cell survival in J14 and JJ012 cells, and vincristine (2 h) and idarubicin (2 h) in H2 and HT1080 cells using clonogenic assays. All four drugs significantly reduced chondrosarcoma cell survival and colony formation in a dose-dependent manner (Figure 7B). However, no differential effects were observed between J14 and JJ012 cells (Figure 7B) or between H2 and HT1080 cells (Figure 3 and Figure 7B), suggesting that their anticancer activity is independent of IDH mutation status.

## 3. Discussion

In this study, we conducted a high-throughput screening of a panel of anticancer compounds in chondrosarcoma cells, many of which have been previously evaluated in preclinical studies or clinical trials. To our knowledge, this is the first analysis to identify potent and synergistic chemotherapeutic agents in IDH1-mutant chondrosarcoma cells. Our screening identified the top 20 most potent compounds, which significantly reduced cell viability across all four cell lines, irrespective of IDH mutation status. These compounds were classified according to their mechanisms of action, including antimitotics, antimetabolites, HDAC inhibitors, antitumor antibiotics/anthracyclines, topoisomerase inhibitors, and a NAE inhibitor. We found that the representative drugs from each class—docetaxel, methotrexate, panobinostat, idarubicin, camptothecin, and pevonedistat— effectively inhibited colony formation, induced apoptosis and cell cycle arrest, and demonstrated synergistic antitumor activity when used in two-drug combinations.

There are limited clinical data related to the efficacy of chemotherapeutics in chondrosarcoma treatment, largely due to the rarity of the disease, which makes recruiting patients for clinical trials challenging. However, some of these agents have been used successfully in other types of bone cancers, such as osteosarcoma and Ewing sarcoma [22]. For instance, methotrexate, doxorubicin, and cisplatin regimens remain standard treatment options for osteosarcoma chemotherapy [23]. While the role of conventional chemotherapy in treating chondrosarcoma remains subject to debate, sparse clinical data show that chemotherapy can be beneficial, particularly in mesenchymal and dedifferentiated chondrosarcoma [4,5]. Italiano et al. reviewed 180 patients treated across 15 institutions in Europe and the United States, reporting a response rate of 31% for mesenchymal chondrosarcoma, 20.5% for dedifferentiated chondrosarcoma, 11.5% for conventional chondrosarcoma, and 0% for clear cell chondrosarcoma [4]. In cases of unresectable conventional chondrosarcoma, retrospective data suggest that systemic therapy is beneficial, with such treatment being associated with a 3-year overall survival (OS) rate of 26% compared to 8% OS in patients who did not receive systemic treatment [24,25]. In line with these findings, several studies have explored the efficacy of the compounds identified in our screening for chondrosarcoma treatment. For instance, a phase II study in which administration of gemcitabine followed by docetaxel in chondrosarcoma patients reported a 14% probability of reaching the target response rate [26]. Additionally, the EUROpean Bone Over 40 Sarcoma Study showed that adding methotrexate to surgery for the treatment of dedifferentiated chondrosarcoma improves survival, although the added benefit of methotrexate compared to other drug regimens was not clear [5]. In our study, we employed the primary central chondrosarcoma cell line, JJ012, and the dedifferentiated chondrosarcoma cell line, HT1080 [12,17]. Our findings, in conjunction with these studies, highlight the potential value of certain conventional chemotherapeutic agents for both subtypes of chondrosarcoma. This underscores the need for further testing of these agents in animal models and clinical trials to better evaluate their therapeutic efficacy.

To better understand the antitumor activities of the selected drugs, we assessed their effects on apoptosis and cell cycle progression in chondrosarcoma cells. The results demonstrated that all six drugs induced apoptosis and cell cycle arrest, primarily in a dose-dependent manner, except for methotrexate, which plateaued at doses of 0.05–0.1 μM. Among these drugs, panobinostat exhibited the most prominent apoptosis-inducing effect, while idarubicin and camptothecin caused the most significant G2/M and S phase arrest, respectively. These complementary effects suggest the potential for synergy when these drugs are used in combination therapies.

In the following synergy analysis, we found that combining panobinostat with idarubicin, pevonedistat, or camptothecin resulted in a significant synergistic effect in chondrosarcoma cells. HDAC inhibitors, such as panobinostat, have been shown to promote cell death, autophagy, apoptosis, or growth arrest in preclinical cancer models. However, these inhibitors are typically less effective as monotherapies, demonstrating greater efficacy when combined with other drugs [27,28]. For example, Bernhart et al. demonstrated that vorinostat, panobinostat, and belinostat reduced cell viability and displayed synergistic effects with doxorubicin in SW-1353 chondrosarcoma cells, thereby increasing the sensitivity of cells to the chemotherapeutic drug [29]. HDAC inhibitors combined with anthracyclines have also been evaluated in clinical trials across various cancer types, including metastatic sarcomas [30,31]. Given that panobinostat regulates epigenetic pathways, it is anticipated that patients with adverse cytogenetic alterations induced by standard chemotherapy may benefit from its addition to treatment regimens.

Additionally, we observed synergistic effects between pevonedistat and camptothecin or methotrexate. Pevonedistat is a first-in-class inhibitor of NAE, which catalyzes the rate-limiting step in protein neddylation—a critical step in the degradation of a wide variety of cellular proteins upstream of the proteasome [32]. Wu et al. demonstrated that pevonedistat activates ER stress-associated proteins, inducing apoptosis and cell cycle arrest in chondrosarcoma cells [33]. Both their study and our findings suggest that targeting NAE-mediated neddylation can suppress chondrosarcoma cell growth, emphasizing the important role of the neddylation pathway in chondrosarcoma progression. The clinical development program for pevonedistat has explored its safety and efficacy, both as a monotherapy and in combination with standard-of-care agents across a range of cancers, including solid tumors and hematological malignancies [34,35,36]. Taken together, the observed synergistic interactions between these compounds highlight their potential for developing combination therapies to improve the treatment of chondrosarcoma.

Lastly, to determine whether depletion of mutant IDH sensitizes chondrosarcoma cells to specific compounds for potential combination therapies with mutant IDH inhibitors, we conducted a drug sensitivity analysis of mutant IDH1 KO cells and compared their responses to those of parental cells. Among the top candidates, floxuridine and dasatinib were identified as the most effective drugs in JJ012-derived IDH1 KO cells, while vincristine and idarubicin were most effective in HT1080-derived IDH1 KO cells. However, clonogenic assays revealed no significant difference in efficacy between parental and KO cells for any of the four drugs. This discrepancy between the clonogenic assay and the standard cell viability assay used in the original screening may be attributed to the different aspects of cell viability assessed by each method. The clonogenic assay measures the ability of cells to proliferate and form colonies, reflecting their long-term reproductive capacity, making it a more stringent test of viability. In contrast, the cell viability assay primarily evaluates membrane integrity, indicating immediate cell viability. As a result, cells may remain metabolically active yet fail to divide or form colonies, which could explain the differences between the two assays. 

In conclusion, our study identifies several highly effective agents that exhibit anti-tumor activity in chondrosarcoma cells, independent of IDH mutation, through the induction of apoptosis and regulation of the cell cycle. Some of these agents also demonstrate synergistic lethality when used in combination. Proof-of-concept studies investigating the effects of these combinatorial treatments on carcinogenesis and metastatic dissemination in animal models will provide valuable insights for the design of rational clinical trials. Based on our findings, we propose that such combination regimens could offer a novel and potentially effective therapeutic approach to the treatment of chondrosarcoma.

## 4. Materials and Methods

### 4.1. Cell Lines and Cell Culture

We utilized two human chondrosarcoma cell lines, JJ012 and HT1080, both of which harbor endogenous IDH1 mutations, as well as their corresponding CRISPR/CAS 9-generated mutant IDH1 KO clones, J14 and H2 [12,17]. Chondrosarcoma cells were cultured in RPMI-1640 medium supplemented with 10% fetal bovine serum (FBS) and 1% penicillin/streptomycin. All cell lines were maintained in a humidified incubator with 5% CO_2_ at 37 °C.

### 4.2. Drug Sensitivity Testing

DST was performed as described previously [21,37,38], and outlined in the workflow in Figure 1. Briefly, cell suspensions were first seeded onto assay plates and exposed to a library of 215 anticancer agents (Table 1). Individual drugs were tested starting at a maximal concentration of 10 μM, followed by a 20,000-fold concentration range. After 72 h of treatment, cell viability was evaluated, and dose–response curves were generated. These curves were analyzed using the DST algorithm to calculate the modified drug sensitivity scoring (DSS_mod_) value for each agent. A significant response (cancer cell-killing) was defined as a DSS_mod_ value ≥5. To assess the selective sensitivity of the mutant IDH KO cells relative to parental cells, a selective drug sensitivity scoring (sDSS_mod_) value was calculated using the following formula: sDSS_mod_ = DSS_mod_ (KO cells)—DSS_mod_ (parental cells). This sDSS_mod_ value integrated efficacy, potency, and therapeutic index for each agent into a numerical metric, which was then used to rank the tumor-specific toxicity.

### 4.3. Cell Survival Analysis by Clonogenic Assay

The clonogenic assay, also known as the colony-formation assay, is an in vitro model that is widely used to assess the survival and proliferative capacity of individual cells. Clonogenic assays were performed as previously described [39]. Briefly, chondrosarcoma cells were treated with individual drugs at varying concentrations for 2 h, 24 h, or 72 h. Following treatment, cells were incubated for 7–10 days at 37 °C to allow for colony formation. After incubation, colonies were fixed with methanol for 10 min, washed three times with PBS, and stained with 10% Giemsa stain (Sigma-Aldrich, St. Louis, MO, USA) for 20 min. The stained colonies were then washed with distilled water and allowed to air dry. Images of the stained colonies were captured using a digital camera (Olympus, Tokyo, Japan).

### 4.4. Apoptosis and Cell Cycle Analysis

Cell apoptosis was evaluated using FITC Annexin V apoptosis detection kit (BD Bioscience, Franklin Lakes, NJ, USA), according to the manufacturer’s protocol. Briefly, cells were washed twice with cold PBS and then resuspended in 1× Binding Buffer at a concentration of 1 × 10^6^ cells/mL. A 100 µL aliquot of the cell suspension was transferred to a 5 mL culture tube, and cells were stained with 5 µL of FITC Annexin and 5 µL of PI for 15 min at room temperature in the dark. Following incubation, 400 µL of 1× Binding Buffer was added to each tube, and the samples were analyzed by flow cytometry within one hour.

For cell cycle analysis, cells were washed with 30–40 mL of PBS and centrifuged at 1000 rpm for 10 min. After resuspending the pellet while vortexing, 5 mL of cold 70–80% ethanol was added dropwise to the pellet and incubated at −20 °C for a minimum of 2 h. To remove ethanol, the cells were washed first with PBS and subsequently with Staining Buffer (BD Bioscience). After centrifugation at 1000–1500 rpm for 10 min, 1 × 10^6^ cells were stained with 0.5 mL of PI/RNase Staining Buffer (BD Bioscience) for 15 min at room temperature. The stained cells were then analyzed by flow cytometry within one hour.

### 4.5. Synergy Analysis

Chondrosarcoma cells were seeded in 96-well plates and treated with the tested compounds at a series of concentrations for 72 h. Cell viability was then assessed by MTS assays (CellTiter 96 ^®^ AQueous One Solution Cell Proliferation Assay, Promega) to determine the cytotoxic effects of these compounds on chondrosarcoma cells. To assess the synergistic effects of drug combinations, we used SYNERGYFINDER 3.0, a web application that is widely used for synergy analysis (https://synergyfinder.fimm.fi accessed on 1 August 2024). The degree of synergy was quantified using the zero interaction potency (ZIP) model, which categorizes drug interactions as synergistic (synergy score > 10), additive (synergy score between −10 and 10), or antagonistic (synergy score < −10) [40].

### 4.6. Statistical Analysis

Statistical analysis was conducted using GraphPad Prism version 8.0 software (GraphPad Software, San Diego, CA, USA). All experiments were performed in triplicate with at least three independent biological repeats, and data are presented as the mean of triplicates ± SEM (standard error of the mean), except for the analysis of total apoptosis, where data are expressed as the mean of triplicates. Differences between groups were analyzed using Student’s t-tests, and *p* values < 0.05 were considered statistically significant.

## Figures and Tables

**Table 1:** Compound library for in vitro drug sensitivity screening.

Class	Compound
**Alkylating Agents**	Bendamustine, Busulfan, Carboplatin, Cisplatin, Cyclophosphamide, Dacarbazine, lfosfamide, lomustine, Methazolastone, Oxaliplatin, Procarbazine, Streptozotocin
**Antimetabolites**	Abitrexate, Adrucil, Azathioprine, Azacitidine, Azaguanine-8, Capecitabine, Carmofur, Cladrabine, Clofarabine, Cytarabine, Decitabine, Febuxostat, Floxuridine, Fludarabine, Fluorouracil, Ftorafur, Gemcitabine, lonidamine, Mercaptopurine, Methotrexate, Nelarabine, Pemetrexed, Thioguanine
**Antimitotics**	10-Deacetylbaccatin, Cephalomannine, Docetaxel, Paclitaxel, Vinblastine, Vincristine
**Antitumor Antibiotics**	Artemether, Azithromycin, Bacitracin, Bleomycin, Hygromycin B, Lincomycin, Methacycline, Ofloxacin, Daunorubicin, Doxorubicin, Epirubicin, ldarubicin
**HDAC Inhibitors**	Belinostat, Panobinostat, Sodium Butyrate, Vorinostat
**Hormone Inhibitors**	2-Methoxyestradiol, Abiraterone, Aminoglutethimide, Anastrozole, Bicalutamide, Clomifene Citrate, Cytaren, Diethylstilbestrol, Doxercalciferol, Enzalutamide, Evista, Exemestane, Flutamide, Fulvestrant, ltraconazole, Letrozole, Megestrol, Mifepristone, Paeoniflorin, Raloxifene, Tamoxifen, Toremifene, Triamcinolone
**lmmunomodulators**	Aspirin, Azathioprine, Betapar, Bindarit, Cortisone, Celecoxib, Dexamethasone, Hydrocortisone, lmiquimod, Maraviroc, Meprednisone, Mizoribine, Mycophenolate, Phenylbutazone, Pimecrolimus, Pomalidomide, Prednisone, Sulindac, Tacrolimus, Thalidomide, Vinpocetine, Zileuton
**Kinase Inhibitors**	Afatinib, Apatinib, Axitinib, Bosutinib, Cabozantinib, Crizotinib, Dasatinib, Erlotinib, lbrutinib, lmatinib, Lapatinib, Masitinib, Nilotinib, Pazopanib, Ponatinib, Regorafenib, Ruxolitinib, Sorafenib, Sunitinib, Tofacitinib, Vandetanib, Vemurafenib
**Proteasome Inhibitors**	Bortezomib, Carfilzomib, Ubenimex
**Rapalogs**	Everolimus, Sirolimus, Temsirolimus
**Topoisomerase** **Inhibitors**	Camptothecin, Etoposide, lrinotecan, Mitoxantrone, Teniposide, Topotecan
**Miscellaneous**	Altretamine, Anagrelide, Bexarotene, Eltrombopag, Geniposide, Hydroxyurea, Mitotane, Pevonedistat (MLN4924), lsotretinoin, Tretinoin
**Other**	Adenine, Adenine hydrochloride, Adenine sulfate, Aprepitant, Atazanavir, Bendamustine, Bepotastine, Bergapten, Blonanserin, Carbazochrome, Clorsulon, Cobicistat, DAPT (GSI-IX), Disulfram, Dorzolamide, Ellagic acid, Epinephrine bitartrate, Esomeprazole, Estradiol, Estrone, Ezetimibe, Famiclovir, Flutamide Flunarizine, Fluvastatin, Gadodiamide, Genistein, Gefitinib, Itraconazole, L-Arginine, Lamotrigine, Leucovorin, Linagliptin, Lenalidomide, Lonidamine, Maraviroc, Medroxyprogesterone, Mesna, Mirabegron, Moroxydine, Mizoribine, Naloxone, Noscapine, Nilvadipine, Pamidronate, Pioglitazone, Ranolazine, Rosiglitazone, Orthovanadate, Temocapril, Teniposide, Tolnaftate, Tolbutamide, Simvastin, Sodium butyrate, Valproic acid, Zoledronic acid, Vismodegib

**Table 2:** The 20 compounds with the highest cancer cell-killing activity across all four chondrosarcoma cell lines, as determined by DSS_mod_ profiling. The representative drugs from each class are highlighted in bold and were selected for further investigation.

Class	Compound	DSS_mod_			
		JJ012	J14	HT1080	H2
Antimitotics	**Docetaxel**	70.9	66.5	51.8	37.6
	Paclitaxel	39	47.8	43.7	34.7
	Vincristine	47.1	45.3	29.1	38.9
	Cephalomannine	28.1	33.8	38	22.7
Antimetabolites	**Methotrexate**	55.6	53.4	46.6	48.9
	Azaguanine	23.7	26.1	28.4	32.8
	Azathioprine	15.4	16.5	24.6	18
	Thioguanine	16.2	14.1	16.7	17.3
	Mercaptopurine	8.3	9	15.1	15.5
HDAC Inhibitors	**Panobinostat**	55	53.5	45.8	50.9
	Belinostat	29.8	26.8	29.4	33.6
	Vorinostat	20	9.8	8.2	12.6
Antitumor Antibiotics	**Idarubicin**	43.9	27.5	31.5	38.4
	Epirubicin	26.9	28.4	34.2	36.9
	Doxorubicin	25	23.5	31.8	35.9
	Daunorubicin	21	20.9	28.5	27.5
Topoisomerase Inhibitors	**Camptothecin**	42	39.2	69.2	67.1
	Topotecan	25.9	24.4	43.5	42.4
	Etoposide	10.5	8.9	18.4	19
NAE Inhibitor	**Pevonedistat** (MLN4924)	17.1	14.6	20.2	20.7

**Table 3:** EC_50_ values of the six representative drugs.

Drug	EC_50_ (µM)			
	JJ012	J14	HT1080	H2
Docetaxel	0.001	0.001	0.001	0.001
Methotrexate	0.018	0.031	0.044	0.038
Panobinostat	0.026	0.036	0.058	0.040
Idarubicin	0.060	0.439	0.235	0.102
Camptothecin	0.080	0.095	0.005	0.006
Pevonedistat (MLN4924)	1.360	2.890	1.096	1.059

## Data Availability

All data supporting the findings of this study are included in the article, and further inquiries can be directed to the corresponding author upon request.
